# 3D Printing Ultraflexible Magnetic Actuators via Screw Extrusion Method

**DOI:** 10.1002/advs.202200898

**Published:** 2022-03-28

**Authors:** Xufeng Cao, Shouhu Xuan, Yinduan Gao, Congcong Lou, Huaxia Deng, Xinglong Gong

**Affiliations:** ^1^ CAS Key Laboratory of Mechanical Behavior and Design of Materials Department of Modern Mechanics University of Science and Technology of China Hefei 230027 China

**Keywords:** 3D print, actuators, magnetic actuation, magnetic materials

## Abstract

Soft magnetic actuators with programmable structure design and controllable deformation ability based on 3D printing technology have attracted extensive attention. In this paper, a novel 3D printing strategy is developed to manufacture the ultraflexible magnetic actuator, in which the printed material is composed of magnetic particles and thermoplastic rubber materials. Different from the traditional fused deposition printing, this printing strategy introduces screw extrusion technology to the heating components of the printer to overcome the problem of filament buckling in the flexible material. Thus, the tensile modulus of the printed products can be reduced to as low as ≈2 MPa. Based on the above method, biomimetic magnetic actuators of the sucker and the pump are constructed for adhering and releasing object and pumping liquid. The contraction performance of the magnetic actuator is studied via a series of experiments and the magnetic field‐induced deformation is analyzed by the multiphysics‐based finite element model. This work proves that ultraflexible magnetic actuators fabricated by this 3D printing strategy show broad prospects in the fields of soft robotics and bionics.

## Graphical abstract

Different from the traditional fused deposition printing, this 3D printing strategy based on the screw extrusion method can effectively overcome the problem of filament buckling in the flexible material. The tensile modulus of printed products can be reduced to as low as ≈2 MPa. Thus, the ultraflexible magnetic actuators fabricated by this 3D printing strategy show broad prospects in the fields of soft robotics and bionics.

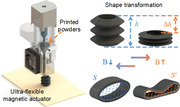

## 1 Introduction

Inspired by natural creatures, the soft actuator with multiple degrees of freedom has attracted extensive attention in realizing rich shape transformation. In this field, a variety of light,^[^ ^1^, ^2^ ^]^ electric,^[^ ^3^, ^4^ ^]^ thermal,^[^ ^5^, ^6^ ^]^ magnetic,^[^ ^7^, ^8^ ^]^ and chemical^[^ ^9^, ^10^ ^]^ stimulus‐response materials have been successfully used to control the shape or motion manipulation of soft actuators. Among these actuation principles, magnetic actuation is widely used in various fields due to its simple control, rapid response and remote contactless actuation.^[^ ^11^, ^12^, ^13^, ^14^, ^15^, ^16^ ^]^ As magnetic stimulus‐response materials, magneto‐active materials consisted of soft matrix and magnetic particles have the ability of fast, untethered, reversible and controllable deformation under the magnetic field.^[^ ^17^, ^18^, ^19^, ^20^, ^21^ ^]^ Among them, soft‐magnetic materials utilize carbonyl iron particles (CIPs) with high saturation magnetization to achieve strong magnetic interaction forces under the actuating magnetic field, resulting in a macroscopic deformation of the soft matrix.^[^ ^22^, ^23^, ^24^ ^]^ Once the magnetic field is removed, the reconfigurable material will return to its original shape due to the inherent elasticity. Thus, the magneto‐active materials can enable the on‐demand magnetic manipulation deformation of actuators, which further adapts to different functional applications, such as adhering, gripping, folding, wireless actuation, pumping and transportation functions.^[^ ^25^, ^26^, ^27^, ^28^, ^29^, ^30^ ^]^

Recently, with the development of additive manufacturing technology, 3D printing technology is gradually used in manufacturing magnetic actuators with complex structures due to the advantages of convenient structure design and rapid prototyping. In order to pursue the versatility and high‐performance of a new generation of magnetic actuators, 3D printing methods based on different magnetic materials are urgently needed. Therefore, with the exploration of printing materials, various magnetic actuators with novel design, complex geometry or programmable structure have gradually been developed through 3D printing strategies including digital light processing (DLP),^[^ ^31^, ^32^, ^33^, ^34^ ^]^ direct ink writing (DIW),^[^ ^35^, ^36^, ^37^, ^38^ ^]^ fused deposition modeling (FDM)^[^ ^39^, ^40^, ^41^ ^]^ and so on.^[^ ^42^, ^43^ ^]^ Based on the magnetic forces or torques generated by the embedded magnetic particles under externally magnetic fields, 3D printed magnetic actuators can be actuated remotely and controlled accurately. Compared with DLP, FDM has the advantages of low cost, no particle sedimentation, simple principle and wide material adaptability. Besides, in the DIW printing process, due to the low modulus of the ink, it is difficult to print the actuators with suspended structures or some curvatures, which will collapse under the force of gravity.^[^ ^44^ ^]^ While the molten material can be cooled rapidly during the FDM printing process, which provides support for subsequently printed structures.

Therefore, the FDM is one of the most attractive printing methods for magnetic actuators with complex structures, which has been successfully applied for biomimetic actuators such as inchworm‐like and flower‐like actuators.^[^ ^40^, ^41^ ^]^ However, limited by the wire‐feeding structure of commercial printers, buckling instability or slipping will occur when the low‐modulus filaments are fed to the extrusion head by drive gear. Therefore, previous research has mainly focused on printing high‐modulus magnetic materials.^[^ ^39^, ^40^, ^41^, ^45^, ^46^, ^47^ ^]^ Whereas, the introduction of screw extrusion technology to the heating components of the printer can effectively solve the problem of continuous feeding in the printing process of soft materials. By using a rotating screw, the melt can be easily transported from the feed hopper to the nozzle.^[^ ^48^, ^49^ ^]^ Moreover, several prototype printers with screw‐based extrusion systems have been explored for the pharmaceutical and food engineering fields.^[^ ^50^, ^51^, ^52^, ^53^ ^]^ Thus, the development of a novel 3D printing strategy based on the screw extrusion device is expected to overcome the problem that only high‐modulus magnetic materials can be printed by the FDM in previous studies.

This work explores a novel 3D printing strategy to fabricate the ultraflexible magnetic actuators, in which the printed material is composed of CIPs and thermoplastic rubber (TPR) materials. Inspired by natural creatures, a sucker and pump actuator are easily constructed with the aid of the 3D printing strategy. The magnetic field‐induced deformation behavior of the hose and pipe actuator is first investigated through the experiments and simulations, and the simulation results match well with the experimental results. Then, the adhesion principle of the sucker actuator and the pumping mechanism of the pump actuator are further demonstrated by conceptual experiments. This novel 3D printing strategy provides new opportunities for the development of ultraflexible magnetic actuators in the field of soft robotics and bionics.

## 2 Results and Discussion

### 2.1 Fabrication and Printing Processes

**Figure 1:**
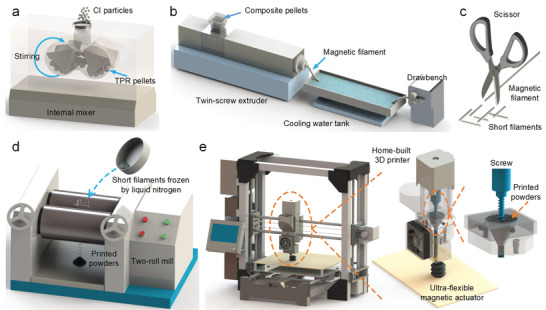
Schematic illustration of a–d) the fabrication processes of printed powders and e) the printing processes of the ultraflexible magnetic actuator.

The fabrication and printing processes of the ultraflexible magnetic actuator were shown in **Figure** **1** (see the Experimental Section for a full description). In brief, the printed powders were fabricated by melt extrusion and liquid nitrogen grinding technique. First, the magnetic composite pellets were fabricated by mixing CIPs and TPR pellets through an internal mixer (Figure 1a). Then, the magnetic filaments were prepared using a twin‐screw extruder (Figure 1b). Next, the resulting materials were cut into centimeter‐sized short filaments by using a scissor (Figure 1c). Subsequently, the short filaments frozen by liquid nitrogen were repeatedly crushed into powders using a two‐roll mill (Figure 1d). The required powders were obtained through a mesh screen. Finally, a home‐built 3D printer based on the screw extrusion method was used to print the ultraflexible magnetic actuators (Figure 1e). It works as follows: the printed powders are first heated to a molten state. Afterward, the screw sheared and delivered the melt to the nozzle which is subsequently deposited onto the print platform. Similar to the fused deposition modeling, the printing quality based on the screw extrusion method is mainly determined by the extrusion temperature, screw speed and printing speed. After optimizing the printing parameters, the feature size is only related to the diameter of the nozzle, which is about 0.6 mm.

### 2.2 Structural and Mechanical Characterization

**Figure 2:**
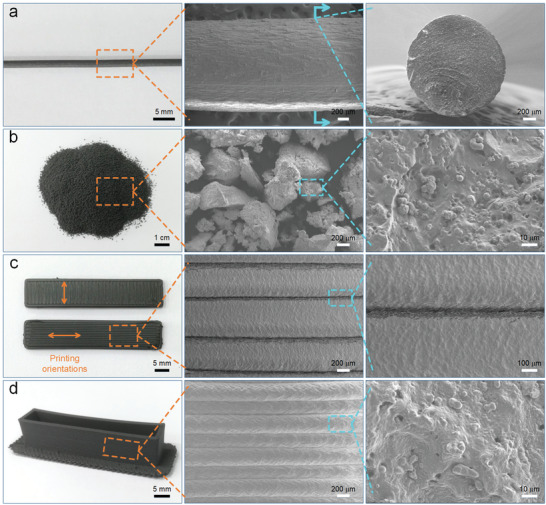
Digital images and SEM microscopy images for a) the magnetic filament, b) the printed powders, c) the individual layer fibers of printed films, and d) the interlayer fibers of the printed frame.

As the required printing material, the magnetic filament was first prepared by melt extrusion. Its digital image and scanning electron microscope (SEM) microscopy image were shown in **Figure** **2**a. Due to the presence of CIPs, the magnetic filament was black. The microscopy image indicated that the magnetic filler was uniformly dispersed in the matrix after melt extrusion and the surface bonding capability between the CIPs and the TPR matrix was very good. Subsequently, the printed powders were fabricated by the liquid nitrogen grinding technique (Figure 2b). The microscopy image showed that the shape of the fine powders after crushing was irregular, and the maximum length of the powders was less than 0.6 mm after sieving. Then, the films with different printing orientations of 0° and 90° were printed (Figure 2c). The surface morphology of the printed films was smooth and the maximum width of the deposition fiber was ≈0.6 mm. Obviously, the adjacent fibers in the individual layer adhered closely together without any voids. Furthermore, the frame was also constructed by layer‐by‐layer deposition (Figure 2d). The microscopy image showed the boundaries between the interlayer fibers along the height direction, which had better surface quality and geometric accuracy than the individual layer fibers. Besides, the combination of CIPs and matrix was consistently well after repeated melt extrusion. In a word, the strong adhesion between the deposition fibers would endow the printed product with good mechanical properties.

**Figure 3:**
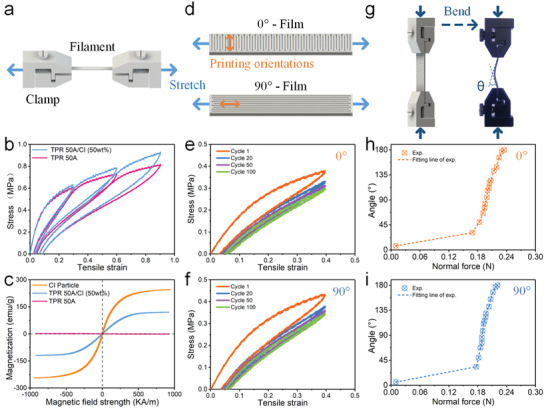
Mechanical characterization. a) Schematic diagram of the tensile test for filaments. b) Tensile stress–strain curves for filaments. c) Magnetization curves of CIPs, magnetic filament and pure TPR filament. d) Schematic diagram of the tensile test for printed films. (Blue arrows: stretching direction, orange arrows: printing orientations.) e,f) Tensile stress–strain curves for printed films with different printing orientations. g) Schematic diagram of the bending test for printed films. h,i) The bending angle versus normal force curves for printed films with different printing orientations.

The tensile test was conducted on the filaments to study the mechanical property (**Figure** **3**a). Figure 3b showed the stress‐strain curves of the filaments under different tensile strains (30%, 60%, and 90%). The elastic modulus and the strength of the magnetic filament were increased compared to pure TPR filament due to the reinforcing effect of the magnetic filler. Moreover, the loading and unloading curves depended on the maximum loading encountered previously, which exhibited a typical Mullins effect. Figure 3c showed the magnetization curves of CIPs, magnetic filament and pure TPR filament, in which the saturation magnetizations were about 245, 123, and 0 emu g^−1^, respectively. It was attributed to the different mass fractions of CIPs within the matrix. The residual magnetization and coercivities were almost zero, which indicated good soft magnetic properties.

A continuous test was carried out to investigate the mechanical performance of the 0° and 90° printed films (Figure 3d). For the 0° and 90° film, the printing orientation was parallel with the direction of width and length, respectively. Figure 3e,f showed the tensile stress‐strain curves under 40% tensile strain for 100 cycles. Obviously, the plastic deformation behavior occurred during the first loading cycle. After multiple loading cycles, the change in the stress‐strain curves gradually became smaller. The tensile modulus of the 0° and 90° films were 1.9 and 2.3 MPa at the linear‐elastic region (*ε* < 5%) during the first loading stage, respectively. After 100 cyclic tensile tests, the stable elastic modulus were 1.4 and 1.6 MPa, indicating a good elastic behavior after slight plastic deformation. The energy dissipation could be represented by the area of the hysteresis curve. The hysteresis curves of the tensile test were almost overlapped after multiple loading, which indicated small energy dissipation, good structure robustness, and good elastic performance. It was attributed to the inherent elasticity of TPR material. Moreover, the mechanical properties were dependent on the deposition directions. The tensile modulus of 0° deposition direction film was slightly lower than that of the 90° film. It was attributed to the more probability of voids and imperfect bonding among the deposited fibers with the larger contact area.

Furthermore, a bending test was carried out to investigate the stability of the deformation of the films (Figure 3g). Due to the limitation of the instrument, various normal forces were applied to the films by adjusting the distance between the clamps with a displacement step increase of 1 mm. Figure 3h,i showed the bending angle of the 0° and 90° films (30  ×  10 × 1.25 mm) as a function of the normal force. During the first loading stage, the bending angle increased slightly but the normal force increased sharply. This was because the bending instability occurred only when the normal force was larger than the load‐carrying capacity of the structure. The result showed that the critical buckling force was slightly lower for the 0° deposition direction film. Consistent with the tensile experiment above, it was because the modulus of the 0° film was slightly smaller. Subsequently, when the load was further increased, the bending angle increased sharply. Although the bending angle and the normal force were positively correlated, the load‐bearing capacity of the structure dropped significantly after bending instability. The slope of the curve was the intuitive characterization of the bending properties of the film, which indicated that the bending properties of 0° and 90° films were comparable. In summary, due to the good contact between deposited fibers, both films had good elasticity and could provide good mechanical property to the structure.

### 2.3 Magnetic Actuation Deformation of the 3D Printed Ultraflexible Magnetic Actuator

**Figure 4:**
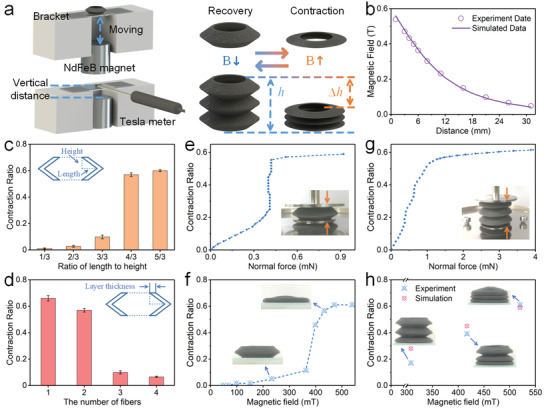
The deformation behavior analysis for the hose actuator. a) The schematic diagrams of the test system. b) The magnetic field versus the distance from the NdFeB magnet. c) Effect of the ratio of length to height on the contraction ratio for the hose unit. d) Effect of the layer thickness on the contraction ratio for the hose unit. e) The contraction ratio versus the normal force for the hose unit. f) The contraction ratio versus the magnetic field for the hose unit. g) The contraction ratio versus the normal force for the hose actuator. h) The contraction ratio versus the magnetic field for the hose actuator. c,d) All the data were obtained by three independent experiments (*n* = 3) and the mean ± standard deviations were presented.

The magnetic field‐induced deformation capability was the most important property of the printed ultraflexible magnetic actuator. To investigate the contraction capability of the hose actuator, the deformation under the various geometric parameters and magnetic fields was investigated. The schematic diagrams of the typical contraction deformation process of the hose actuator under the magnetic field were shown in **Figure** **4**a. The contraction ratio could be calculated by measuring the height change of the actuator. It was defined as φ=Δh/h, where *h* and Δ*h* were the initial height and the change value of the height, respectively. Various magnetic fields were applied to the actuator by adjusting the distance between the NdFeB magnet (30  ×  30 mm) and the actuator. Besides, the applied magnetic field was measured at the center of the bottom of the actuator by using a Gauss mete, which showed nonlinear growth as the magnet approached closely (Figure 4b).

The contraction capability of the actuator depended on both geometric parameters and magnetic fields. The ratio of length to height could be employed to indicate the folding degree of the hose unit. Obviously, keeping the same magnetic field, increasing the ratio of length to height generated a larger contraction ratio (Figure 4c). The contraction ratio increased from 0.01 to 0.6 with the folding degree increased from 1/3 to 5/3. Besides, the contraction ratio was further compared on actuators with different layer thicknesses. When the number of fibers increased from 1 to 4 (thickness: 0.8 to 3.2 mm), the contraction ratio decreased from 0.66 to 0.07 (Figure 4d). The enhanced rigidity was attributed to the decreased folding degree and increased layer thickness, which weakened the effect of the magnetic field on the deformation.

Moreover, the influence of the magnetic field on the contraction capability was also studied. Here, static loading was first employed to evaluate the compressive performance of the actuator (Figure 4e). The hose unit was placed on the stage of the rheometer. The force sensor was employed to measure the normal force under a series of different loading displacements. In the low range of force, the contraction ratio increased linearly with the normal force. When the normal force reached 0.4 mN, the structure unit became unstable and lost load‐bearing capacity, and the contraction ratio had a sharp increase from 0.25 to 0.55. Then, upon further increasing the normal force, the contraction ratio remained unchanged due to the compacted structure. Figure 4f showed the contraction process of the hose unit under various magnetic fields. Consistent with the above compress experiment, the curves could be divided into three stages. The contraction ratio was slightly increased under the small magnetic field. When the magnetic field was large enough, the magnetic force acting on the hose unit increased significantly with the increasing of the deformation due to the magneto‐elastic coupling. The nonlinearly increased magnetic interaction force caused the structure to continually compress down until the structure was nearly overlapped.

However, the magnetic field‐induced deformation was a bit different for the hose actuator. In the static loading experiment, the contraction ratio remained positively correlated with the normal force until the structure compacted, which exhibited a stable equivalent compression stiffness (Figure 4g). Furthermore, owing to the interaction between the magnetic particles and the magnetic field, the hose actuator could quickly contract under the magnetic field. At the initial stage, the hose actuator remained straight. When the applied magnetic field was 302 mT, the bottom unit of the actuator was first attracted and contracted. It was attributed to the nonlinear increasing of the magnetic field around the magnet, which induced the sharp increasing of the magnetic interaction force. Then, with the further increasing of the magnetic field, the entire hose actuator kept contracting layer by layer until it contracted completely. During the shrinking process, the stable contraction ratios of the actuator were 0.39 and 0.61 under the magnetic fields of 433 and 539 mT, respectively (Figure 4h). When the magnetic field was removed, the entire actuator would recover to its original state due to the inherent elasticity of the material. In addition, the reduction of contraction ratio was negligible after deforming for 100 cycles (Figure S1, Supporting Information), which showed good repetitive deformation performance.

**Figure 5:**
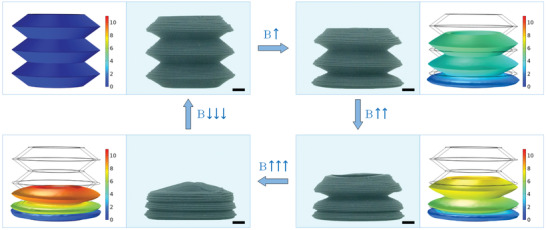
The magnetic field‐induced deformation and finite element simulation of the hose actuator (scale bar: 2 mm).

Furthermore, the magnetic field‐induced deformation process of the hose actuator was studied by finite element analysis (FEA). Upon the applied magnetic field, the hose actuator contracted due to the magnetic interaction force. **Figure** **5** showed the displacement distribution of the hose actuator under the various magnetic field. Here, the simulated results were very close to the experimental results at higher magnetic field (Figure 4h), indicating that the simulation model could predict the deformation behavior of the actuator. Unfortunately, there existed some disparities at the low magnetic field case, which was caused by that the finite element model could not accurately simulate the instability process. Video S1 (Supporting Information) showed the deformation process of the hose actuator and the above results showed that the hose actuator had a rapid, stable, and accurate shape transformation response.

**Figure 6:**
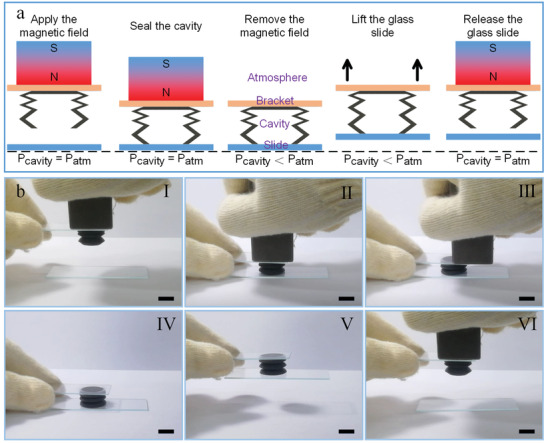
Demonstration experiment for the sucker actuator. a) Schematic diagram of the design and principle for the sucker actuator. b) Snapshots of sequential grasping and releasing the glass slide with sucker actuator (scale bar: 10 mm).

Here, the actuator prepared by the novel 3D printing strategy was used in the further application. Inspired by the suction cups of the octopus tentacle, the sucker actuator could be constructed by sealing one end of the hose actuator. **Figure** **6**a showed the schematic of the sucker actuator through integrated printing molding, which consisted of the upper membrane and the lower hose actuator. The upper membrane was fixed on the bracket with glue. This unique structure could realize the adhesion and release functions, in which the deformation of the structure was actively controlled by the applied magnetic field. The flexibility of the materials ensured close contact with the smooth surface of the object to form a seal. Specifically, before the contact, the magnetic field was applied to generate the contraction deformation, which reduced the cavity volume and stored the elastic energy. In this state, the pressure in the cavity was the same as the atmospheric pressure (i.e., *P*_cavity_ = *P*_atm_). Then, the magnetic field was removed after the contact between the actuator and the target surface. The structure tended to return to the original shape due to the elastic deformation energy. Besides, the volume of the cavity was then increased by lifting the actuator, which also reduced the cavity pressure. It would result in a high adhesion due to the negative pressure difference between the cavity pressure and the atmospheric pressure (*P*_cavity_ < *P*_atm_). When the magnetic field was applied to the actuator again, the negative pressure difference decreased or even reduced to zero due to the contraction deformation, resulting in low adhesion (*P*_cavity_ = *P*_atm_). At this state, the actuator would release the object.

Due to the poor sealing between the sucker actuator and the glass slide, the structure would recover to its initial shape with the air leakage into the cavity over time (Figure S2, Supporting Information), resulting in the releasing of the glass slide. In order to reveal the principle of the release caused by magnetic deformation, a short‐time operation experiment was selected to ensure that the negative pressure in the cavity was basically unchanged. Figure 6b briefly showed the manipulation process of the sucker actuator to demonstrate its availability (Video S2, Supporting Information). First, the sucker actuator with shrink deformation was moved downward to contact with the glass slide (Figure 6b, I). Then, the actuator was pressed lightly to ensure the seal between the actuator and the glass slide (Figure 6b, II). When the magnetic field was removed, the contractive actuator tended to recover to the initial shape due to the initial elasticity, which generated the negative pressure difference between the cavity and atmosphere (Figure 6b, III and IV). Subsequently, through the effect of the pressure difference, the glass slide was successfully lifted by the actuator (Figure 6b, V). When the magnetic field was applied again to provide the same magnetic interaction force, the actuator contracted to the same shape as before, thereby generating a low adhesion state. Finally, the glass slide was successfully released (Figure 6b, VI), which demonstrated the potential adhesion and release capability of the sucker actuator. Subsequently, the demonstration experiment with grasping and releasing the table tennis (Figure S3, Video S3, Supporting Information) was also performed, which demonstrated the actuator could also be used for catching curved structures with smooth surfaces. Moreover, the structural strength of the actuator was strong enough to lift glass sheets with a weight of 24.5 g (Figure S4, Supporting Information). However, due to the poor sealing, the adhesive duration decreased sharply with the increasing of the weight, which was less than 1 s at the maximum weight. These concept demonstration experiments provided a promising strategy for the design and operation of the intelligent grabber and facilitated the potential application of the 3D printed ultraflexible magnetic actuator in soft robotics.

**Figure 7:**
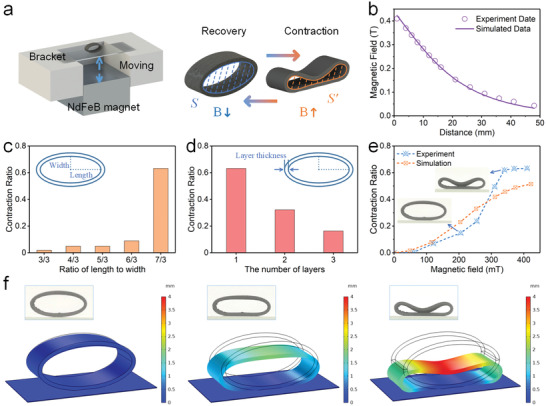
The deformation behavior analysis for the pipe actuator. a) The schematic diagrams of the test system. b) The magnetic field versus the distance from the NdFeB magnet. c) Effect of the ratio of length to width on the contraction ratio. d) Effect of the layer thickness on the contraction ratio. e) The contraction ratio versus the magnetic field. f) The magnetic field‐induced deformation and finite element simulation.

Due to the advantages of 3D printing technology, the ultraflexible magnetic actuator with different structures could be easily constructed. Here, the magnetic field‐induced deformation capability of the pipe actuator under the various geometric parameters and magnetic fields was also investigated. The schematic diagrams of the typical deformation behavior of the pipe actuator under the magnetic field were shown in **Figure** **7**a. The contraction ratio was defined as $\varphi =\left(S-S^{\prime }\right)/S$, where *S* and *S*′ were the original area and the final area, respectively. By adjusting the distance between the NdFeB magnet (50 × 50 × 30 mm) and the actuator, different magnetic field strength could be obtained at the center of the bottom of the actuator (Figure 7b), which also showed the nonlinear growth of the field strength as the distance shortened.

To study the influence of the geometric parameters on the magnetic field‐induced deformation, the pipes with different ratios of length to width were tested under the same magnetic field (Figure 7c). Obviously, the pipes with small ratio of length to width had slight contraction deformation. The contraction ratio increased from 0.02 to 0.09 as the ratio of length to width increased from 3/3 to 6/3. It could be explained that the structure with large ratio of length to width beared greater bending moment under the same magnetic field. When the ratio of length to width was 7/3, the contraction ratio increased sharply to 0.63. It was because that the structure would lose stability when the magnetic field was large enough. Then, the contraction ratio was further compared on actuators with different layer thicknesses. When the number of fibers increased from 1 to 3 (thickness: 0.8 to 2.4 mm), the contraction ratio decreased from 0.63 to 0.16 (Figure 7d). Compared with the thick‐walled pipe, the thin‐walled pipe had greater deformation. The weaker deformation was attributed to the increasing flexural rigidity of the structure with the layer thickness.

Furthermore, the influence of the magnetic field on the contraction deformation of the pipe was investigated. Figure 7e exhibited the contraction process of the pipe under different magnetic fields. The curves could be divided into three stages. When the external magnetic field was less than 60 mT, the structural deformation could be ignored. As the magnetic field increased, the contraction ratio began to increase significantly. Obviously, when the magnetic field increased from 205 to 340 mT, the contraction ratio had a sharp increase from 0.15 to 0.62. Then, it remained unchanged with the magnetic field further increasing. This was because the magnetic force acting on the structure increased with the shrink deformation, and when the magnetic force was large enough, the upper wall of the structure exhibited bistable deformation. The closer the upper wall to the magnet, the stronger the magnetic field. Hence, the contraction ratio increased faster and faster until the upper wall contacted the lower wall.

Moreover, the magnetic field‐induced deformation response of the pipe was simulated by the finite element analysis. Figure 7f showed the representative contraction deformation simulation, which was corresponded to the above experiment. Obviously, the shrink deformation of the pipe increased with the increasing of the magnetic field. The simulated results had a similar variation tendency to the experimental data (Figure 7e), indicating that the finite element model could describe the deformation behavior to a certain extent.

**Figure 8:**
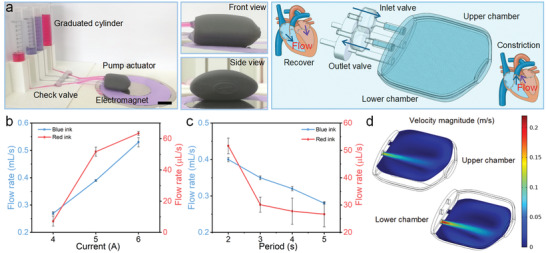
Demonstration experiment for the pump actuator. a) The digital image and schematic diagram of the pump actuator (scale bar: 2 cm). b) Plot of flow rate versus current. c) Plot of flow rate versus period. d) The simulated results for the fluid velocity of the cross section. b,c) All the data were obtained by three independent experiments (*n* = 3) and the mean ± standard deviations were presented.

The pipe could be extended into a pump structure by printing structures with different cross‐section sizes. The prototype design of the pump was inspired by the heart structure (**Figure** **8**a). The basic concept was composed of 3D printed pump chambers for liquid pumping, commercial check valves for backflow prevention and connecting pipelines. The connections were coated with hot melt adhesive to prevent leakage during testing. To improve the actuation efficiency, the time‐varying magnetic field was generated by the electromagnet. When the magnetic field was applied, the top section of the pump was deformed and contracted by the magnetic force, while the bottom section contracted due to the squeezing between the structure and the electromagnet. In this case, the deformation of the top part was smaller than that of the bottom part. The asymmetric deformation would generate different positive pressures concomitantly, which closed the inlet check valve and opened the outlet one, pumping the fluid in it through the check valve. In the demonstration experiment, water mixed with red and blue dyes was added to the upper and lower chambers, respectively. Under cyclic loading of the magnetic field, the liquid in the inlet was rapidly transferred to the outlet (Video S4, Supporting Information). The flow rate could be calculated by the pumping time and the volume change of the liquid in the graduated cylinder, and the test was repeated three times.

The influence of the magnetic field and the loading period on pumping efficiency was studied. The magnetic field of 443, 525, and 582 mT could be obtained at the center of the bottom section of the pump under 4, 5, and 6 A current, respectively. The current with the period of 2, 3, 4, and 5 s was used in the electromagnet, in which the power‐on time was half of the period. Obviously, the flow rate increased with the increasing of the magnetic field (Figure 8b). When the current change from 4 to 6 A with a period of 2 s, the flow rate increased from 0.27 to 0.53 mL s^−1^ and 7.14 to 63.4 µL s^−1^, respectively. Moreover, upon the applied magnetic field of 525 mT, the flow rate increased from 0.28 to 0.4 mL s^−1^ and 26.7 to 51.7 µL s^−1^ with the increasing of the frequency, respectively (Figure 8c). Hence, the flow rate was determined by both the magnetic field strength and the loading period. Furthermore, to visualize the fluid flow in the pump, a finite element model was established. After solving the multiphysics coupling, the fluid velocity distribution in the cross section of the upper and lower chamber under 5 A current could be obtained (Figure 8d). As expected, the velocity near the outlet area was maximum. Similar to the pumping experimental results, the fluid velocity in the lower chamber was greater than that in the upper chamber. Therefore, the intuitive notion of the pumping performance for the actuator could be qualitatively described with the aid of the simulation model.

## 3 Conclusion

In summary, this work developed a novel 3D printing strategy based on the FDM method and the screw extrusion technology to manufacture ultraflexible magnetic actuators. To this end, printed powders consisting of CIPs and TPR pellets were first developed, which could be printed on the home‐built printer. Then, the tensile and bending experiments demonstrated that the film structure had good elasticity and mechanical properties, in which the tensile modulus and the critical buckling force of 0° deposition direction film were slightly lower than the 90° film. Next, the ultraflexible hose and the pipe actuator were printed to demonstrate the controllability of the magnetic field‐induced deformation, and the contraction performance of actuators depended on both geometric parameters and magnetic fields. Furthermore, related deformation processes had been also studied by finite element simulation. Finally, functionalized manipulations of the magnetic actuator were verified by the controllable deformation of the sucker and pump actuator for adhering objects and pumping liquid. This research proved that the ultraflexible magnetic actuator fabricated by this 3D printing strategy has broad application prospects in the field of soft robotics, allowing complex structural design and controllable deformation ability.

## 4 Experimental Section

### Materials

The CIPs (7 µm average diameter) used as the magnetic filler were purchased from BASF in Germany. As the flexible matrix material, the TPR (50A shore hardness) was supplied in pellets form by the Hanwha TOTAL Petrochemical Co., Ltd. Before processing, the TPR pellets and the CIPs were dried overnight at 50 ℃.

### Fabrication and Printing Processes

First, the magnetic composite pellets were fabricated by mixing CIPs and TPR pellets with the ratio of 1:1 through an internal mixer at 60 °C (Figure 1a). Then, the magnetic filaments with CIPs content of 50 wt% were prepared using a twin‐screw extruder (Figure 1b), in which the CIPs were uniformly dispersed within the TPR matrix by melt‐blending. The magnetic composite pellets were transported from the feeding hopper down to the extrusion nozzle under the processing temperature of 180 °C. The molten mixture was pulled away from the die with a drawbench and cooled down with water to fabricate filaments. Next, the resulting materials were cut into centimeter‐sized short filaments by using a scissor (Figure 1c). Subsequently, the short filaments frozen by liquid nitrogen were repeatedly crushed into powders using a two‐roll mill (Figure 1d). The fine powders with a length of less than 0.6 mm were obtained through a 30 mesh screen and dried overnight at room temperature. Finally, a home‐built 3D printer based on the screw extrusion method was used to print the ultraflexible magnetic actuators (Figure 1e). The printer was mainly composed of a drive motor, an extrusion screw, a hopper structure, heat components and a cooling system. The motor shaft was connected with the screw through the rigid sleeve coupling. Here, the extrusion screw adopted a self‐tapping screw of standard parts (5 mm diameter and 80 mm length), which was convenient for the miniaturization of the printers and the replacement of screw parts. Small screw pitch was also beneficial to improve the accuracy of the melt delivery, while finer printed powder was required. Besides, some threads at the front end of the screw were removed to exhaust the air from the melt, which could maintain a constant flow of melt material. Furthermore, the combination of agitator arm on the coupling and funnel‐shaped hopper could be used to achieve continuous feeding of powder. Finally, the printer structure was removable for easy maintenance and replacement of printing materials. The printer driver adopted the open source Marlin firmware, which could easily set the firmware parameters. The Simplify3D software was used to set printing parameters: printing temperature of 225 °C, printing rate of 6 mm s^−1^, nozzle diameter of 0.6 mm, layer height 0.27 mm, and infill density of 100%.

### Characterization and Instruments

The microscopic morphology of the printed powders and the samples was characterized by scanning electron microscopy (SEM, GeminiSEM 500, ZEISS). The magnetic hysteresis loop of materials was measured by using a Hysteresis Measurement of Soft and Hard Magnetic materials (HyMDC Metis, Leuven, Belgium). The uniaxial tension, bending, and compression test were conducted by a dynamic mechanical analyzer (DMA, ElectroForce 3200, TA Instruments, USA) and a commercial rheometer (Physical MCR 301, Anton Paar Co., Austria). The magnetic field was measured by a Tesla meter (HT20, Shanghai Hengtong Magnetic Technology Co., Ltd., China). Furthermore, the 3D printing process was completed by using a home‐built 3D printer.

### Numerical Methods

The magnetic field‐induced deformation and pump performance of the ultraflexible magnetic actuators were analyzed by the commercial software COMSOL Multiphysics 5.5. The geometric models including air, solid, and fluid domain were designed with SolidWorks 2016 and directly imported into COMSOL Multiphysics. The FEA for the magnetic actuators involved multiple physical fields, so the FEA model based on magnetic field module, solid mechanics module, and laminar flow module was established. The FEA included three steps: the calculation of the magnetic field distribution, the analysis of the magnetic field‐induced deformation under the previous magnetic field, and the research of the pump performance affected by the solid domain. The magnetic force on the actuator was imported from the force calculation interface in the magnetic field module, which was calculated by the integration of Maxwell surface stress tensor. After that, in the solid mechanics module, the actuator bottom was set as a fixed constraint. The boundary load (magnetic force) was applied to the surface of the actuators. In the laminar flow module, the boundary condition was set to constant outlet pressure. Besides, Young's modulus, relative permeability and density of the materials were used as input for the simulation. Finally, after proper meshing and solving, the simulation results could be obtained.

### Statistical Analysis

Data in Figures 4c,d and 8b,c were presented with mean ± standard deviations. The sample size (*n*) for each statistical analysis was 3 unless otherwise indicated. The results were tested for difference using *t*‐test with OriginPro2017 software. A level of *p* < 0.05 was considered to be statistically significant.

## Conflict of Interest

The authors declare no conflict of interest.

## Supporting information

Supporting Information

Supplemental Video 1

Supplemental Video 2

Supplemental Video 3

Supplemental Video 4

## Data Availability

The data that support the findings of this study are available from the corresponding author upon reasonable request.
