# Supplementary material for: 3D Printing Ultraflexible Magnetic Actuators via Screw Extrusion Method
Source: Adv Sci (Weinh). 2022 Mar 28;9(16):2200898. doi: 10.1002/advs.202200898 (PMC9165489; doi:10.1002/advs.202200898)
Supplement: Supplementary file 1 — Supporting Information [file ADVS-9-2200898-s003.pdf]

Supporting Information

3D Printing Ultra-flexible Magnetic Actuators *via* Screw Extrusion Method

Xufeng Cao, Shouhu Xuan, Yinduan Gao, Congcong Lou, Huaxia Deng, Xinglong Gong**

CAS Key Laboratory of Mechanical Behavior and Design of Materials, Department of Modern Mechanics, University of Science and Technology of China, Hefei 230027, China
E-mail: gongxl@ustc.edu.cn (XL Gong); xuansh@ustc.edu.cn (SH Xuan).

Supplementary Figures

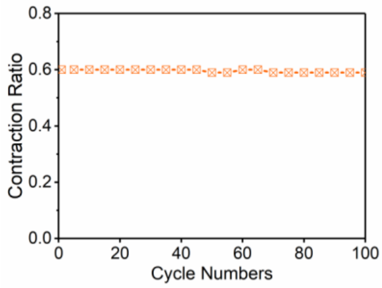

Figure S1. Cycle test of the contraction deformation performance.

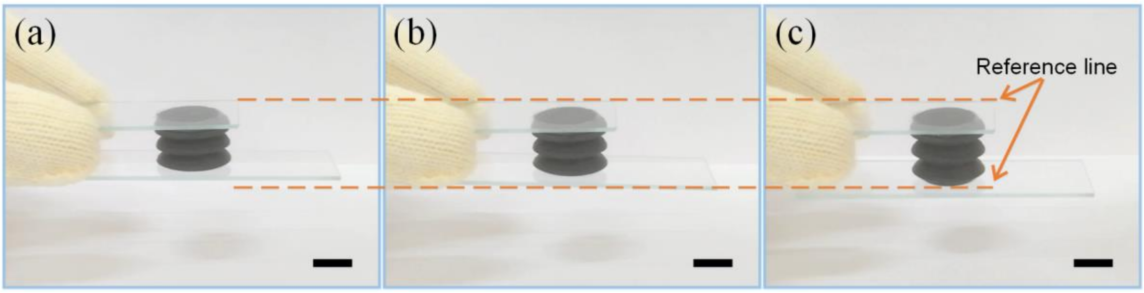

Figure S2. (a-c) Digital images of the deformation process with the air leakage. (Scale bar: 10 mm)

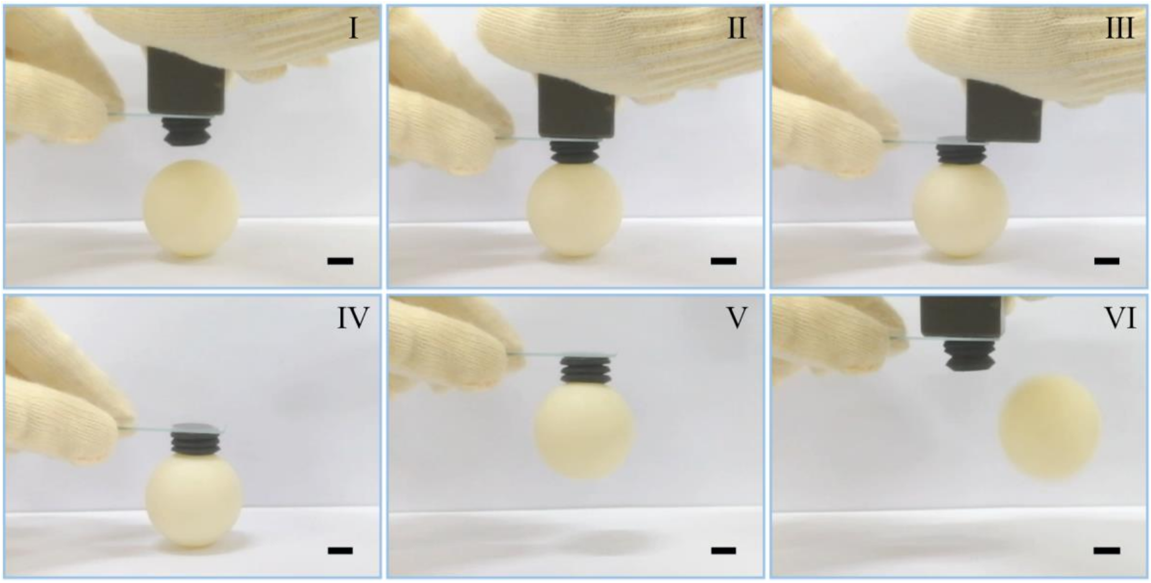

Figure S3. Snapshots of sequential grasping and releasing the table tennis with sucker actuator. (Scale bar: 10 mm)

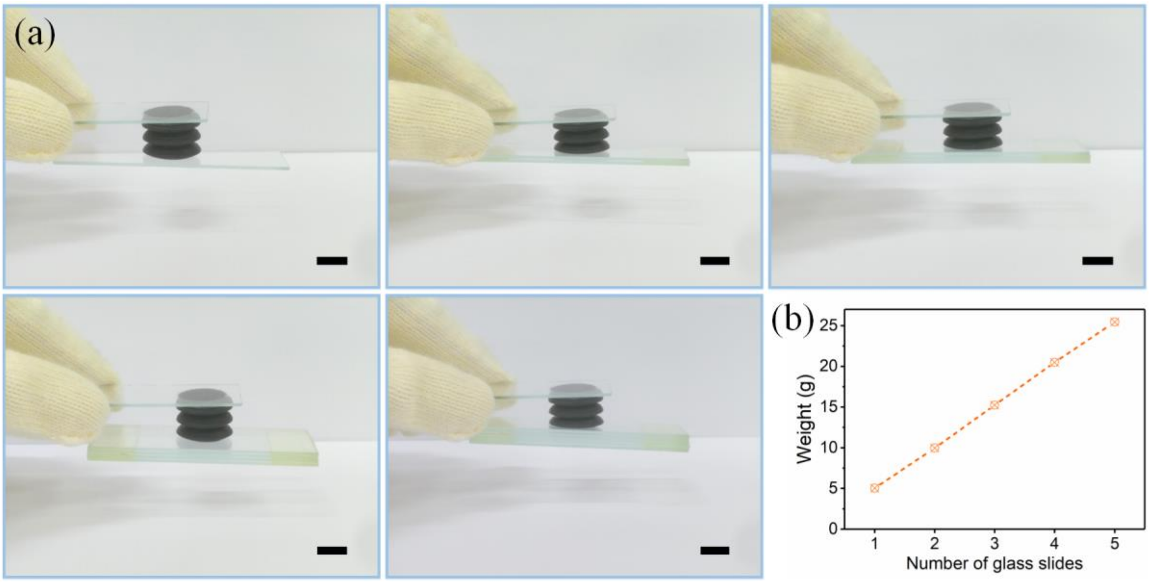

Figure S4. (a) Digital images of grasping the glass slides (Scale bar: 10 mm). (b) The weight versus the number of glass slides.
